# Complement system component dysregulation is a distinctive feature of COVID-19 disease: a prospective and comparative analysis of patients admitted to the emergency department for suspected COVID-19 disease

**DOI:** 10.1007/s11239-021-02617-x

**Published:** 2021-12-14

**Authors:** Nadine Gauchel, Marina Rieder, Krystin Krauel, Isabella Goller, Maren Jeserich, Ulrich Salzer, Ana Cecilia Venhoff, Niklas Baldus, Luisa Pollmeier, Luisa Wirth, Winfried Kern, Siegbert Rieg, Hans-Jörg Busch, Maike Hofmann, Christoph Bode, Daniel Duerschmied, Achim Lother, Lukas A. Heger

**Affiliations:** 1 Department of Medicine III (Interdisciplinary Medical Intensive Care), Medical Center, Faculty of Medicine, University of Freiburg, Freiburg, Germany; 2 Department of Cardiology and Angiology I, Heart Center, University of Freiburg, Hugstetter Strasse 55, 79106 Freiburg, Germany; 3 Department of Rheumatology and Clinical Immunology, University Hospital of Freiburg, Freiburg, Germany; 4 Institute of Experimental and Clinical Pharmacology and Toxicology, Faculty of Medicine, University of Freiburg, Freiburg, Germany; 5 Division of Infectious Diseases, Department of Medicine II, Medical Center, Faculty of Medicine, University of Freiburg, University of Freiburg, Freiburg, Germany; 6 Department of Emergency Medicine, Faculty of Medicine, University Hospital of Freiburg, University of Freiburg, Freiburg, Germany; 7 Department of Medicine II, Faculty of Medicine, University Hospital Freiburg, University of Freiburg, Freiburg, Germany

**Keywords:** COVID-19, Complement components, Coagulopathy, Von Willebrand factor, Calprotectin

## Abstract

The complement system (CS) plays a pivotal role in Coronavirus disease 2019 (COVID-19) pathophysiology. The objective of this study was to provide a comparative, prospective data analysis of CS components in an all-comers cohort and COVID-19 patients. Patients with suspected COVID-19 infection admitted to the Emergency department were grouped for definite diagnosis of COVID-19 and no COVID-19 accordingly. Clinical presentation, routine laboratory and von Willebrand factor (vWF) antigen as well as CS components 3, 4 and activated 5 (C5a) were assessed. Also, total complement activity via the classical pathway (CH50) was determined. Levels of calprotectin in serum were measured using an automated quantitative lateral flow assay. We included 80 patients in this prospective trial. Of those 19 (23.7%) were tested positive for severe acute respiratory syndrome coronavirus 2 (SARS-CoV-2). Patients with COVID-19 had higher levels of CS components 5a and 4 (54.79 [24.14–88.79] ng/ml vs. 35 [23.15–46.1] ng/ml; p = 0.0433 and 0.3772 [± 0.1056] g/L vs. 0.286 [0.2375–0.3748] g/L; p = 0.0168). COVID-19 patients had significantly higher levels of vWF antigen when compared to the control group (288.3 [± 80.26] % vs. 212 [151–320] %; p = 0.0469). There was a significant correlation between CS C3 and 5a with vWF antigen (r_s_ = 0.5957 [p = 0.0131] and r_s_ = 0.5015 [p = 0.042]) in COVID-19 patients. There was no difference in calprotectin plasma levels (4.786 [± 2.397] µg/ml vs. 4.233 [± 2.142] µg/ml; p = 0.4175) between both groups. This prospective data from a single centre all-comers cohort accentuates altered levels of CS components as a distinct feature of COVID-19 disease. Deregulation of CS component 3 and C5a are associated with increased vWF antigen possibly linking vascular damage to alternative CS activation in COVID-19.

**Supplementary Information:**

The online version contains supplementary material available at 10.1007/s11239-021-02617-x.

## Highlights

- Complement system (CS) component dysregulation is a distinctive feature of COVID-19 disease.
- Patients with COVID-19 have higher levels of CS components 5a and 4.
- Elevated levels of CS component 5a correlate with elevated levels of von Willebrand Factor antigen in COVID-19 patients.
- Calprotectin plasma levels do not differ between COVID-19 patients and emergency department all-comers.

## Introduction

The complement system (CS), as part of the innate immune response, plays a profound role in coordinating the inflammatory response to pathogens and its unrestrained activation has been implicated in the pathogenesis of Coronavirus disease 2019 (COVID-19) [1–3]. Multiple studies have shown increased levels of CS components in patients with COVID-19 as well as deposition of activated complement proteins in injured organs [3, 4]. Recently a severe acute respiratory syndrome coronavirus 2 (SARS-CoV-2) binding site affecting a CS activating serine-protease was discovered as possible target for therapy [5]. Understanding CS activation in COVID-19 could provide insights into the pathogenesis of hypercoagulability and increased risk for coagulopathic events, both hallmarks of COVID-19 disease [4, 6–8].

The relevance of COVID-19-coagulation abnormalities remains of pivotal importance as a substantial proportion of patients with mild COVID-19 is developing, sometimes unrecognized, thromboembolic complications [9]. More specific targets are needed to optimize treatment strategies. Currently several inhibitors of the CS are in clinical trials for COVID-19 treatment (C3: NCT04395456; C5: NCT04355494 and C5a: NCT04346797) [10].

The CS is activated via different pathways [10–12]. One of this pathways seems to be via endothelial cell dysfunction and injury [13]. Von Willebrand factor antigen has been suggested as marker of endothelial dysfunction in vascular diseases before and imbalance of the VWF-axis has been implicated in COVID-19 disease [14, 15].

Several other markers are suggested for COVID-19 prognosis and diagnosis as well [16]. Calprotectin a member of the S100 family has recently gained increasing attention as a potential novel biomarker of inflammatory disorders [17]. Despite lacking consensus on measurement of calprotectin levels several studies report significant differences in calprotectin plasma levels in COVID-19 disease [18, 19]. However, little is known of the diagnostic capacities of calprotectin in emergency department patients during the COVID-19 pandemic.

## Methods

### Study design

We here report data from an investigator-initiated, single-center prospective registry study to evaluate biomarkers associated with COVID-19 (DRKS00021206, Deutsches Register klinische Studien (DRKS)) conducted at the University Medical Center–University of Freiburg.

The protocol of this study conforms to the ethical guidelines of the 1975 Declaration of Helsinki and was approved by the institutional ethical committee of the University of Freiburg (EK 153/20).

### Study population

All-comers admitted to the department of emergency medicine of the University Medical Center–University of Freiburg between 26th of March 2020 and the 22th of May 2020 with suspected COVID-19 were included in this prospective trial. The decision to perform a PCR-test for severe acute respiratory syndrome coronavirus 2 (SARS-CoV-2) was made independently of study inclusion by the treating physician and patients were asked to participate before the test results were available.

Written informed consent was obtained from all patients prior to study inclusion.

**Fig. 1:**
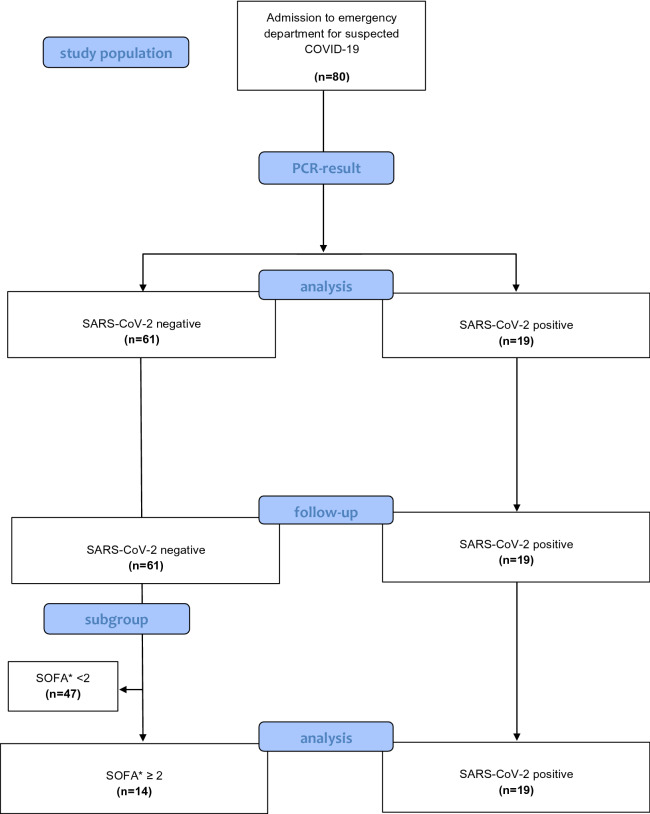
Schematic representation of the allocation to the positive or negative group of the 80 participants. The flow diagram is based on the template of the CONSORT flow diagram. (38) *SOFA Sequential Organ Failure Assessment

Patients with a positive PCR-test for SARS-CoV-2 were finally allocated to the “positive” COVID-19 group, patients with a negative PCR-test for SARS-CoV-2 to the control group (Fig. 1). No included patient was vaccinated against SARS-CoV-2.

### Study plan

If patients had agreed to participate, overall characteristics such as sex, age, body-mass-index (BMI), medical history, laboratory parameters, clinical symptoms or previous medication were recorded.

### Endpoint

The primary end-point was diagnosis of COVID-19.

### Complement system components

The concentration of complement factors C3 and C4 in serum were determined by nephelometry on an Atellica NEPH 630 System (Siemens Healthcare GmbH, Erlangen, Germany) using standard settings and assays as recommended by the manufacturer. Semiquantitative assessment of functional classical complement pathway activation (CH50) in serum was performed with the Wieslab Complement system Classical pathway ELISA kit (SVAR LIFE SCIENCEAB, Malmö, Sweden), following the instructions given by the manufacturer.

Component C5a level in serum was determined by a commercially available ELISA kit (DuoSet, R&D Systems), according to the manufacturer’s instructions.

### Calprotectin

Calprotectin (MRP8/14 or S100A8/A9) in serum was measured using Quantum Blue sCAL reagents (BÜHLMANN Laboratories AG, Schönenbuch, Switzerland) on a Quantum Blue Reader 2nd generation (BÜHLMANN Laboratories AG, Schönenbuch, Switzerland) using standard settings and protocols as recommended by the manufacturer.

### von Willebrand factor & Serotonin

Von Willebrand factor antigen and activity were measured using Sysmex CS-5100 System™ (Siemens Healthcare Diagnostics, Erlangen, Germany) according to the manufacturer’s instructions. Reagents used on the CS-5100/ analyzers were INNOVANCE VWF Ac/Standard Human Plasma/ Control Plasma N, Control Plasma P (Catalogue No.: OPHL03 Siemens Healthcare Diagnostics Products GmbH) for vWF activity and von Willebrand Antigen/ Standard Human Plasma/ Control Plasma N, Control Plasma P (Catalogue No.: OPAB03 Siemens Healthcare Diagnostics Products GmbH) for vWF antigen.

Serotonin was analyzed in serum samples using an enzymatic immunoassay kit (Serotonin ELISAFastTrack, LDN, Nordhorn, Germany) according to the manufacturer’s instructions. All samples and standards were analyzed on a microplate reader set at 450 nm.

### Data analysis

For analysis, data were blinded to patient identity. Statistical analyses were performed using SPSS (version 25, IBM, SPSS Statistics, Armonk, USA) and GraphPad Prism 9 (GraphPad Software, San Diego, USA).

Variables following Gaussian distribution were compared using student’s t-test, non-normally distributed continuous values by using Mann–Whitney-U test. Categorical variables were assessed by chi-square test or Fisher’s exact test as appropriate.

A two-tailed p-value less than 0.05 was considered statistically significant. Simple linear regression analysis was used to model the relationship between two complement components and von Willebrand Factor.

Data are presented as mean ± standard deviation if found to follow a Gaussian distribution or otherwise as median with interquartile range. Spearman's correlation for non parametric data was used to measure the strength and direction of monotonic association between CS components and vWF antigen.

### Follow-up

Patients were contacted 1 month after discharge to assess whether they had persistent incapacity to work.

## Results

### Baseline characteristics

We included 80 patients admitted to ED for respiratory failure in this prospective cohort study. Of those 19 (23.7%) were tested positive for SARS-CoV-2 and henceforth allocated to the COVID-19 group. In the COVID-19 cohort, 50,8% were male and in the control group 40% (p = 0,4). Patients in the non-COVID-19 control group were age matched (62.9 [± 19.6] years vs. 58.9 [± 14.2] years; p = 0.4074) with the COVID-19 group. In the non-COVID-19 group, 4 (6.6%) had symptomatic anemia, 9 (14.8%) patients had pneumonia, 9 (14.8%) patients were hospitalized for cardiac decompensation, 4 (6.6%) had pulmonary embolism, 16 (26.2) patients hat urogenital or gastrointestinal infections and 11 (18%) various forms of cancer (including fever in neutropenia). (Supplement Fig. 1).

Patients with COVID-19 had similar Body Mass Index when compared to the control group (24.54 [21.9–27.17] kg/m^2^ vs. 25.9 [± 5.1] kg/m^2^; p = 0.4414).

There was no significant difference in history of coronary artery disease (13.1% vs 10%; p = 0.133), hypertension (50.1% vs. 30%; p = 0.1048) or diabetes mellitus (14.8% vs. 10%; p = 0.5902) between the control group and COVID-19 patients. COVID-19 patients were more likely to be on angiotensin II receptor blockers (35% vs. 13.3%; p = 0.0316). (Table 1) Nobody in the COVID-19 group and 4 (6%) patients in the control-group were on medication with potassium-sparing diuretics.

**Table 1:** Baseline characteristics at admission

Variable	Non-COVID 19 (n = 61)		COVID-19 (n = 19)		P-value
Age (years)	62.9	(± 19.6)	58.9	(± 14.2)	0.4074^a^
Body Mass Index	24.54	(21.9—27.17)	25.9	(± 5.1)	0.4414^b^
Mean arterial pressure (mmHg)	99	(± 16.2)	99.3	(± 11.84)	0.9413^a^
Heart Rate (beats/minute)	88	(± 19.8)	90.2	(± 15.7)	0.6769^a^
Respiratory rate/minute	23	(18.3–30)	25.3	(± 9.1)	0.8893^b^
O2-Saturation (%)	96	(94–98)	96	(93.598)	0.7234^b^
Temperature (in °C)	37.5	(± 0.99)	37.5	(± 0.83)	0.846^a^
SOFA* Score	1	(0–2)	0.5	(0–1.75)	0.6926^b^
Leukocytes (Tsd/µl)	8.9	(6—13.6)	4.7	(± 2.31)	< 0.0001^b^
Haemoglobin (g/dl)	11.41	(± 2.78)	12.7	(± 2.5)	0.0691^a^
Creatinin (mg/dl)	0.97	(0.77–1.4)	0.92	(0.72–1.2)	0.3405^b^
C-reactive protein (mg/l)	29.30	(4.52–79.9)	46.8	(± 56.8)	0.405^b^
Procalcitonin (ng/ml)	0.08	(0.05–0.35)	0.07	(0.06–0.15)	0.5378^b^
Aspartat-Aminotransferase (U/I)	25	(18.8–37)	34.5	(28.5–69.8)	0.0046^b^
PTT at admission (sec.)	31	(28–34)	29.8	(± 3)	0.2947^b^
Laktatdehydrogenase (U/I)	234.5	(192–295.5)	249	(191–341)	0.4543^b^
Creatinkinase (U/I)	68.5	(48.8–119.5)	100.3	(± 62.6)	0.6168^b^
D-Dimere	1.15	(0.34–2.13)	1.13	(0.47–1.98)	0.9087^b^
Calprotectin (µg/ml)	4.786	(± 2.397)	4.233	(± 2.142)	0.4175 ^a^
Pro-BNP** (pg/ml)	318	(81.5–3247)	106	(50–404)	0.0211^b^
Length of hospital stay (days)	7	(2.75–15)	12.25	(± 9.8)	0.2217^b^
Sex (Male in %)	50.8		40		0.4^c^
Visual analogue scales (VAS)	4.6	(± 2.8)	5.4	(± 2.9)	0.3489^a^
Cough (%)	37.7		65		0.033^c^
Dyspnea (%)	57.4		35		0.0822^c^
Fever (%)	49.2		80		0.0158^c^
Angiotensin II receptor blockers (%)	13.3		35		0.0316^c^
Coronary Artery Disease (%)	13.1		10		0.7133^c^
Hypertension (%)	50.1		30		0.1048^c^
Diabetes Mellitus (%)	14.8		10		0.5902^c^
History or current smoking (%)	52.5		50		0.8485^c^

*SOFA Sequential Organ Failure Assessment (SOFA) Score, **BNP B-type natriuretic peptide; Variables following Gaussian distribution were compared using student’s t-test (**a**), non-normally distributed continuous values by using Mann–Whitney-U test (**b**). Categorical variables were assessed by chi-square test or Fisher’s exact test as appropriate (**c**)

### Laboratory data & characteristics at admission

COVID-19 patients were more likely to present with fever (80% vs. 49.2%; p = 0.0158), cough (65% vs. 37.5%; p = 0.033) with a tendency for dyspnoea (57.4% vs. 35%; p = 0.0822) when compared to non-COVID-19 patients. There was no difference in the Visual Analogue Scales (VAS) for subjective disease severity between both groups (4.6 [± 2.8] vs. 5.4 [± 2.9]; p = 0.3489). At admission there was no statistical difference between the control group and patients with COVID-19 in respect to mean arterial pressure (99 [± 16.2] mmHg vs. 99.3 [± 11.84] mmHg; p = 0.9413), heart rate (88 [± 19.8] beats/minute vs. 90.2 [± 15.7] beats/minute; p = 0.6769), respiratory rate (23 [18.3–30] per minute vs. 25.3 [± 9.1] per minute; p = 0.8893) or difference in saturation at admission (96 [94–98] % vs 96 [93.5–98]; p = 0.7234).

There was no statistically significant difference in severity of disease assessed by SOFA Score between patients with COVID-19 and the control group (0.5 [0–1.75] vs. 1 [0–2]; p = 0.6926).

Non-COVID-19 patients had higher blood levels of leukocytes (8.9 [6–13.6] K/µl vs. 4.7 [± 2.31] K/µl; p = < 0.0001) and pro-BNP (318 [81.5–3247] pg/ml vs. 106 [50–404] pg/ml; p = 0.0211) when compared to COVID-19 patients. There was no difference in C-reactive protein (29.30 [4.52–79.9] mg/l vs. 46.8 [± 56.8] mg/l; p = 0.405) or procalcitonin (0.08 [0.05–0.35] ng/ml vs. 0.07 [0.06–0.15] ng/ml; p = 0.5378) between the control-group and the COVID-19 group.

COVID-19 patients had significantly higher blood levels of aspartat-aminotransferase when compared to the control group (34.5 [28.5–69.8] U/I vs. 25 [18.8–37] U/I; p = 0.0046).

There was no difference between calprotectin plasma levels between patients of the control group and patients with COVID-19 (4.786 (± 2.397) µg/ml vs. 4.233 (± 2.142) µg/ml; p = 0.4175).

### Complement & complement activation

**Fig. 2:**
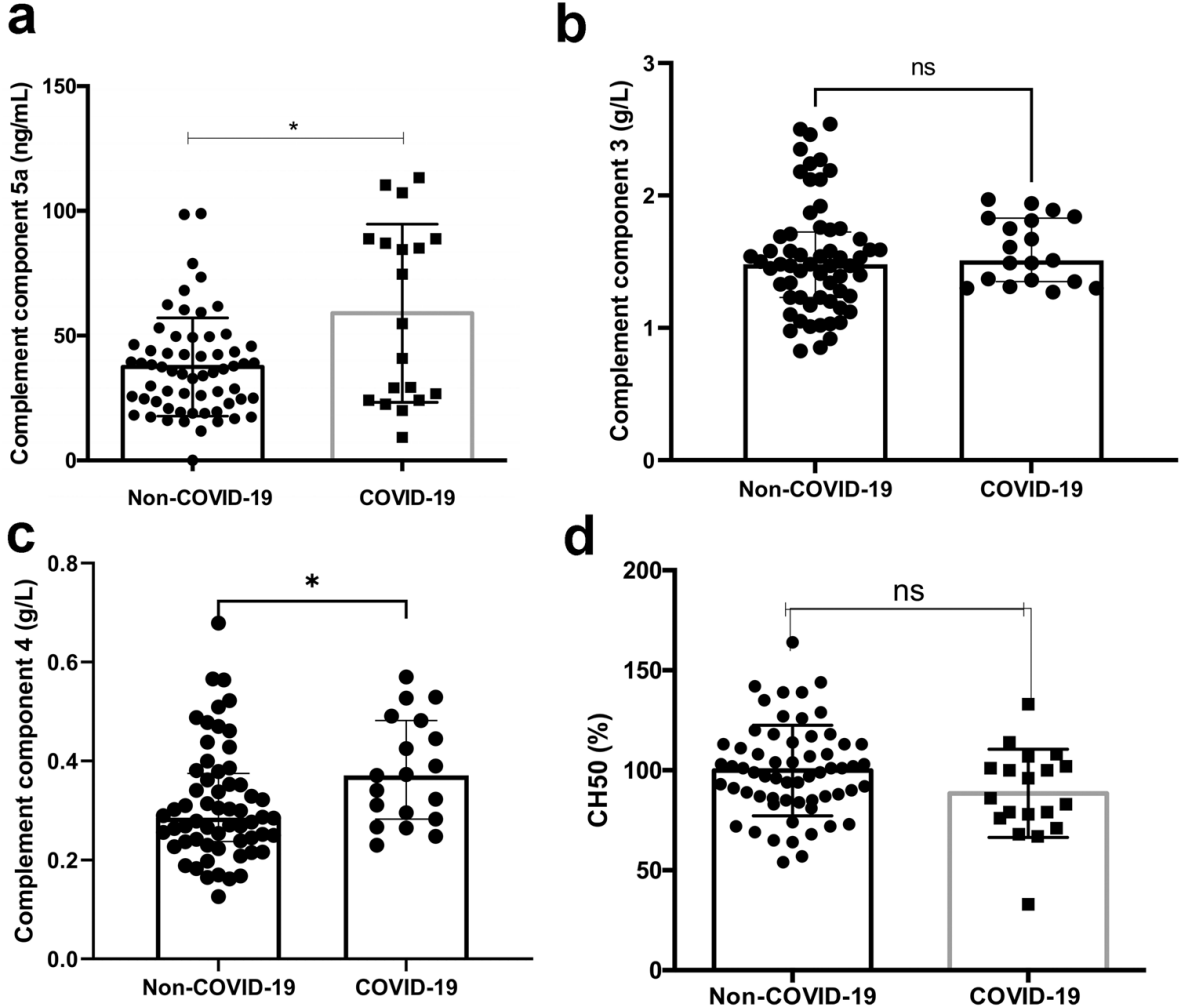
Comparative analysis of Complement factors (**a** Complement component 5a in ng/ml; **b** Complement component 3 in g/L; **c** Complement component 4 in g/L;** d** CH50 (%) in patients hospitalized for respiratory failure and COVID-19 respectively. Data are presented as scatter block with median and interquartile range

Patients with COVID-19 had statistically significant higher levels of complement components 5a and 4 (54.79 [24.14–88.79] ng/ml vs. 35 [23.15–46.1] ng/ml; p = 0.0433 and 0.3772 [± 0.1056] vs. 0.286 [0.2375–0.3748]; p = 0.0168). There was no significant difference in classical CS activation or CS C3 levels between COVID-19 patients and the control group (CH50: 88.47 [± 22.1] % vs. 99.85 [± 22.6] %; p = 0.06; C3: 1.51 [1.35–1.83] g/L vs. 1.53 [± 0.4252] g/L; p = 0.3049). (Fig. 2).

**Fig. 3:**
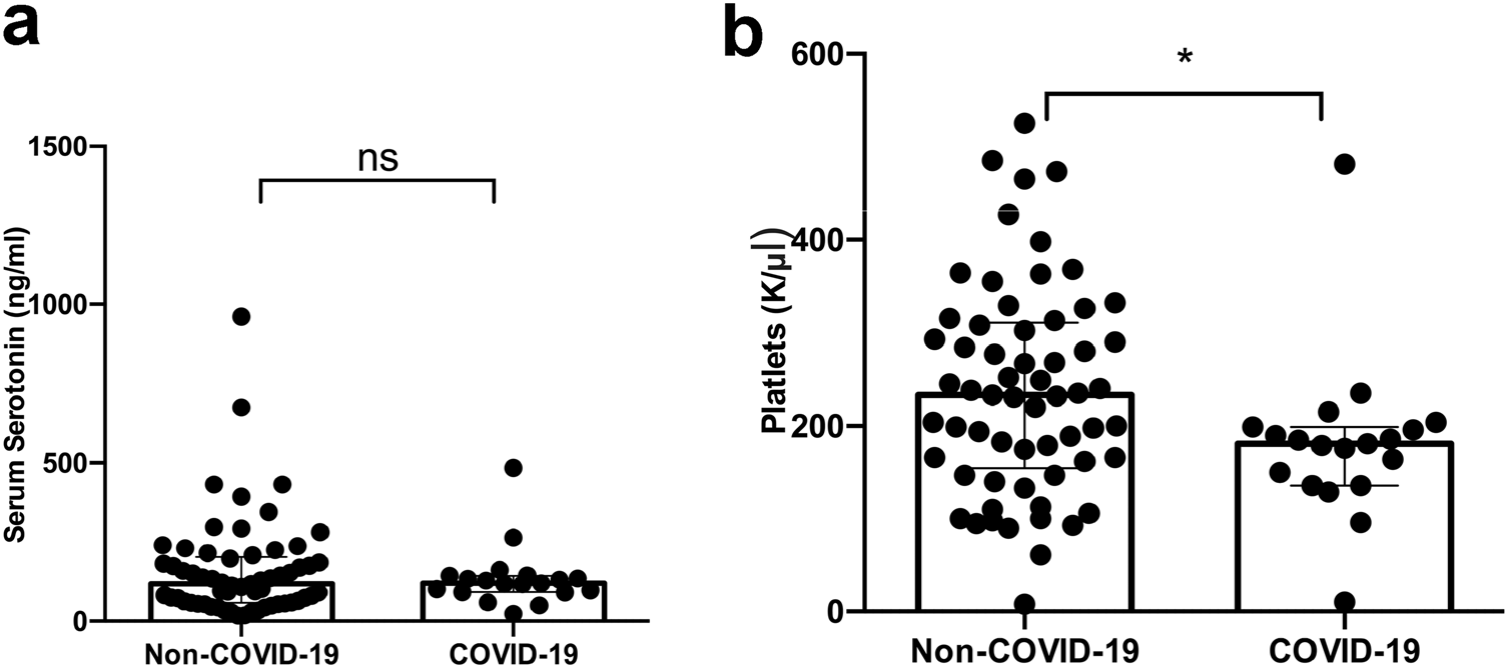
Comparative analysis of plasma serotonin **a** and platelet count **b** in patients hospitalized for respiratory failure and COVID-19 respectively. Data are presented as scatter block with median and interquartile range

Patients with COVID-19 had lower platelet count when compared to the control group (180 [136–198.3] K/ul vs. 237.1 [± 91.73] K/ul; p = 0.0304). There was no statistically significant difference in serotonin plasma levels between the COVID-19 cohort and the control group (109.5 [± 35.98] ng/ml vs. 116 [58.6–201.3] ng/ml; p = 0.6). (Fig. 3).

**Fig. 4:**
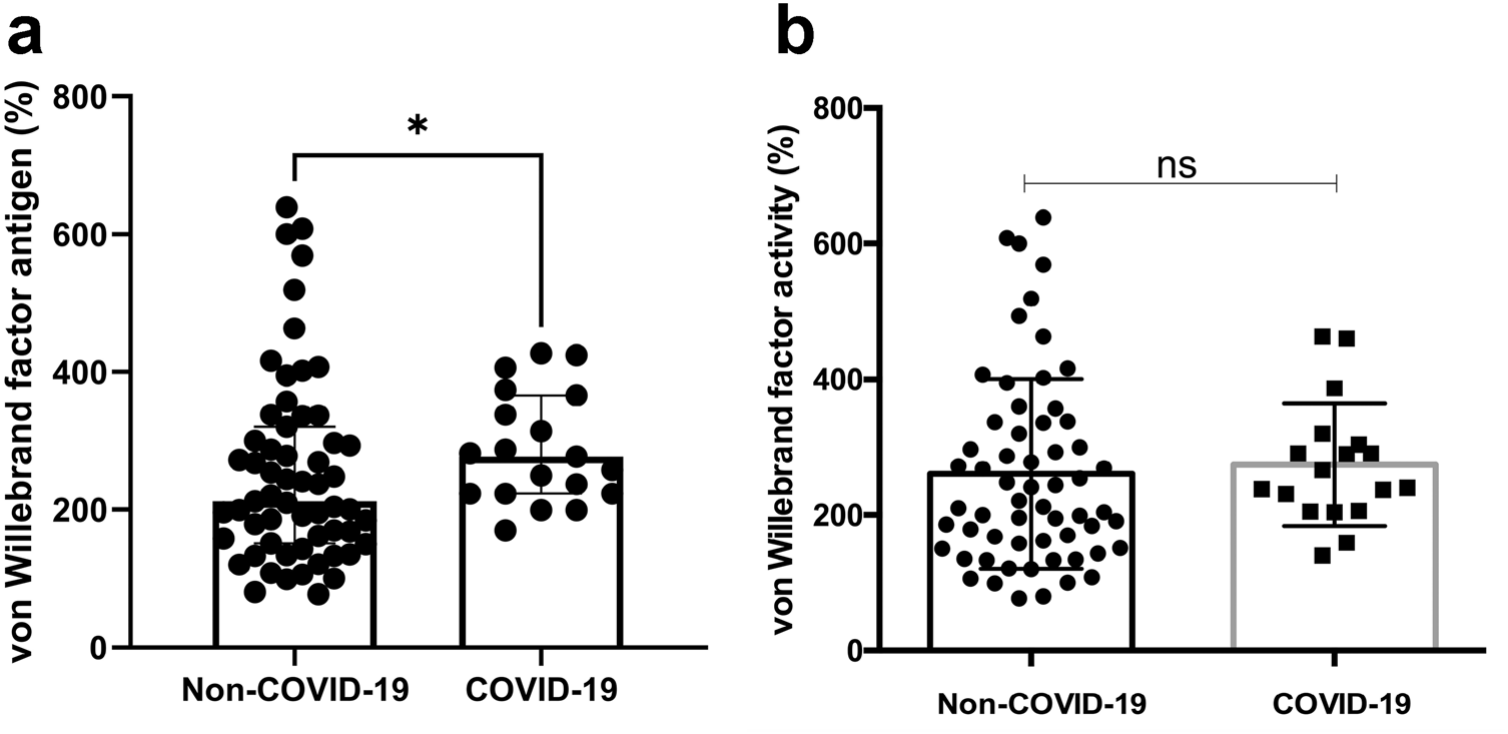
Comparative analysis of plasma levels of von Willebrand factor antigen **a** and von Willebrand factor activity **b** in patients hospitalized for suspected COVID-19 and definite diagnosis of COVID-19 respectively. Data are presented as scatter block with median and interquartile range

Patients with COVID-19 had significantly higher levels of von Willebrand Factor antigen when compared to the control group (288.3 [± 80.26] % vs. 212 [151–320] %; p = 0.0469). There was no difference in von Willebrand factor activity between the control group and COVID-19 patients (147 [± 129.6] % vs. 166 [106–220] %; p = 0.0469). (Fig. 4).

There was a significant correlation between CS C3 and 5a with vWF antigen (r_s_ = 0.5957 [p = 0.0131] and r_s_ = 0.5015 [p = 0.042]) in COVID-19 patients.

In a subgroup analysis comparing non-COVID-19 patients with SOFA Score ≥ 2 (n = 14) to patients with COVID-19 statistical analysis showed increased levels of complement component 3 (1.321 [± 0.2804] g/l vs. 1.51 [1.35–1.83] g/l; p = 0.031), 4 (0.2673 [± 0.0797] g/l vs. 0.3772 [± 0.1056] g/l; p = 0.0027) and 5a (0.159 [0.1175–0.203] pg/ml vs. 0.493 [0.139–0.583] pg/ml; p = 0.0378). (Supplement Fig. 2).

### Characteristics of hospital stay & follow-up

There was no statistically significant difference in length of hospital stay(7 [2.75–15] days vs. 12.25 [± 9.8] days; p = 0.2217) between the control group and COVID-19 patients. None of COVID-19 patients and 6 (9.8%) of non-COVID-19 patients were referred to the intensive care unit. In patients with COVID-19, 10 (52.6%) patients were employed prior to hospitalization as against 12 (19.7%) patients in the non-COVID-19 control group. At one-month follow-up, significantly more patients from the COVID-19 cohort were still incapacitated for work (70% vs. 25%; p = 0.0348).

## Discussion

In this single centre prospective real world data analysis we evaluate the CS in COVID-19 disease in respect to an all comers cohort admitted to the ED. Our data implicate a derailed CS with overexpression of CS components in COVID-19 patients plasma as a distinctive feature of COVID-19 disease [20].

Several studies have described the crosstalk between CS and coagulation in sepsis [21]. Our data add to the amounting evidence, that the pro-thrombotic state in COVID-19 disease is in a similar more profound way fuelled by a derailed CS [2–4, 21, 22]. Interestingly our data shows C4 and C5a elevation but no difference in C3 expression. This could be in line with recent experimental data suggesting a "C3 bypass" activation of C5 by surface-deposited C4 [23].

In clinical practice, especially activated CS component 5a is considered to be a critical determinant of neutrophil recruitment and activation in thrombosis and early dampening could have the potential to reduce leukocyte accumulation, thrombus initiation and propagation [10, 24, 25]. However, mechanisms mediating C3a/C5a generation during a pro-thrombotic state are poorly understood. Endotheliopathy as a hallmark of septic patients with injured endothelial cells provides a scaffold for coagulation [26]. Correspondingly, we report increased plasma levels of von Wilebrand factor antigen as biomarker pointing to vascular injury in COVID-19 patients [13, 27, 28]. This increase in vWF antigen was associated with CS components linking vWF antigen to CS alteration.

Correspondingly, recent data show, that complement activation and endothelial perturbation parallel COVID-19 severity [29]. Our prospective data from an all-comers cohort accentuates vWF antigen as a distinct feature of COVID-19 and add to the amounting evidence linking activation of CS with vWF antigen and endothelial damage [12, 13]. Several publications indicate alternative pathways of CS activation in COVID-19 to be predominant [29, 30]. Platelets are an important modulator of CS and secrete serotonin upon activation [31, 32]. With the majority of peripheral serotonin stored in platelets, we hypothesized that activation of platelets and subsequent clearance from the blood stream could be the cause of COVID-19 thrombocytopenia and would result in increased plasma levels of serotonin. However, this was not the case. This could be partially explained by recent evidence showing, that increased platelet activation is a hallmark of mainly severe COVID-19 [33]. Nevertheless, platelets are chief effector cells in thrombosis and our data confirm a deregulation in platelet homeostasis in COVID-19. The exact mechanism of platelets in the pathogenesis of COVID-19 however, remains elusive.

Several studies have evaluated biomarkers that can help predict severe complications in COVID-19. Recently, calprotectin a member of the S100 family has gained increasing attention as a potential novel biomarker of inflammatory disorders [17]. Despite lacking consensus on measurement of calprotectin levels several studies report significant differences in calprotectin plasma levels in COVID-19 [18, 19]. Our prospective data analysis showed no difference in calprotectin levels at admission. Whether calprotectin may serve as predictor of disease progression is yet to be established.

Baseline laboratory parameters are suggested in COVID-19 diagnosis because of their cost effectiveness and easy accessibility. Interestingly, we show no difference in D-Dimers between the two cohorts at admission [34]. This is in line with recent evidence suggesting dynamic changes in D-Dimers over the course of hospitalization to predict subsequent coagulopathy in COVID-19 patients [35].

Recently a meta-analysis isolated a pattern of abnormal liver enzymes in COVID-19 [36]. Our data confirm, that intensive monitoring for liver injury may be needed in cases with COVID-19.

While there was no subjective difference in Vision Analogue Scale for disease severity at admission and no difference in length of hospital stay our data clearly indicate the relevance of the post-acute COVID-19 syndrome and its influence on the economic fallout of the pandemic with an increased proportion of COVID-19 patients being incapacitated for work after one month. Also we show, that typical symptoms of COVID-19 including cough, fever and dyspnoea are reliable and decisive in ED triage and can help to optimize patient flow especially with increasing numbers of hospital admission [37].

## Limitations

We report data from a single centre study. Larger clinical trials are needed. Complement levels are very dynamic and are result of constant synthesis and decay and especially for activation markers like C5a the preanalytical handling (= the quality of the collected sample) is crucial. While we tried to establish immediate analysis blood sample collection was part of the routine process and therefore subject to inter-individual differences. Also, only a small fraction of all CS factors have been quantified by us. There are many others, some of which may be also increased or decreased in COVID-19 patients and still correlate with the disease status. Finally, since we did not collect cycle thresholds of initial SARS-CoV-2 polymerase chain reaction tests of included patients at the time point of collecting the blood sample, patients could be in different stages of the disease.

## Conclusion

This prospective comparative data from a single centre all-comers cohort accentuates dysregulation of CS components as a distinct marker for COVID-19 disease. Highlighting the importance of alternative CS activation pathways through endothelial damage we show an association of CS components and von Willebrand factor antigen in COVID-19 patients. Our data provides evidence, that despite deregulation in platelet homeostasis leading to thrombocytopenia in COVID-19 patients, serum levels of serotonin are not increased. Also we show, that calprotectin, a promising marker of disease progression is of limited use in COVID-19 assessment in emergency evaluation. Finally, our data adds to the understanding of the socio-ecological fallout of the COVID-19 pandemic showing a higher rate of prolonged incapacity for work in post-COVID-19 patients.

## Supplementary Information

Below is the link to the electronic supplementary material.

Supplementary file1 (DOCX 220 KB)

## Abbreviations

COVID-19: Coronavirus disease 2019
CS: Complement System
ED: Emergency department
SARS-CoV-2: Severe acute respiratory syndrome coronavirus 2
VWF: Von Willebrand factor

## References

[1] Dunkelberger JR, Song WC (2010). Complement and its role in innate and adaptive immune responses. Cell Res.

[2] Java A, Apicelli AJ, Liszewski MK, Coler-Reilly A, Atkinson JP, Kim AH (2020). The complement system in COVID-19: friend and foe?. JCI Insight.

[3] Ma L, Sahu SK, Cano M, Kuppuswamy V, Bajwa J, McPhatter JN (2021). Increased complement activation is a distinctive feature of severe SARS-CoV-2 infection. Sci Immunol.

[4] Magro C, Mulvey JJ, Berlin D, Nuovo G, Salvatore S, Harp J (2020). Complement associated microvascular injury and thrombosis in the pathogenesis of severe COVID-19 infection: A report of five cases. Transl Res.

[5] Gao T, Hu M, Zhang X, Li H, Zhu L, Liu H, et al. Highly pathogenic coronavirus N protein aggravates lung injury by MASP-2-mediated complement over-activation. medRxiv. 2020; 25: 777

[6] Ackermann M, Verleden SE, Kuehnel M, Haverich A, Welte T, Laenger F (2020). Pulmonary vascular endothelialitis, thrombosis, and angiogenesis in Covid-19. N Engl J Med.

[7] Noris M, Benigni A, Remuzzi G (2020). The case of complement activation in COVID-19 multiorgan impact. Kidney Int.

[8] Tan CW, Tan JY, Wong WH, Cheong MA, Ng IM, Conceicao EP (2021). Clinical and laboratory features of hypercoagulability in COVID-19 and other respiratory viral infections amongst predominantly younger adults with few comorbidities. Sci Rep.

[9] Fan BE, Umapathi T, Chua K, Chia YW, Wong SW, Tan GWL (2021). Delayed catastrophic thrombotic events in young and asymptomatic post COVID-19 patients. J Thromb Thrombolysis.

[10] Campbell CM, Kahwash R (2020). Will complement inhibition be the new target in treating COVID-19-related systemic thrombosis?. Circulation.

[11] Willems E, Alkema W, Keizer-Garritsen J, Suppers A, van der Flier M, Philipsen R (2019). Biosynthetic homeostasis and resilience of the complement system in health and infectious disease. EBioMedicine.

[12] Feng S, Liang X, Kroll MH, Chung DW, Afshar-Kharghan V (2015). von Willebrand factor is a cofactor in complement regulation. Blood.

[13] Bonaventura A, Vecchie A, Dagna L, Martinod K, Dixon DL, Van Tassell BW (2021). Endothelial dysfunction and immunothrombosis as key pathogenic mechanisms in COVID-19. Nat Rev Immunol.

[14] Mancini I, Baronciani L, Artoni A, Colpani P, Biganzoli M, Cozzi G (2021). The ADAMTS13-von Willebrand factor axis in COVID-19 patients. J Thromb Haemost.

[15] Horvath B, Hegedus D, Szapary L, Marton Z, Alexy T, Koltai K (2004). Measurement of von Willebrand factor as the marker of endothelial dysfunction in vascular diseases. Exp Clin Cardiol.

[16] Danwang C, Endomba FT, Nkeck JR, Wouna DLA, Robert A, Noubiap JJ (2020). A meta-analysis of potential biomarkers associated with severity of coronavirus disease 2019 (COVID-19). Biomark Res.

[17] Mahler M, Meroni PL, Infantino M, Buhler KA, Fritzler MJ (2021). Circulating calprotectin as a biomarker of COVID-19 severity. Expert Rev Clin Immunol.

[18] Garcia L, de Guadiana R, Mulero MDR, Olivo MH, Rojas CR, Arenas VR, Morales MG (2021). Circulating levels of GDF-15 and calprotectin for prediction of in-hospital mortality in COVID-19 patients: A case series. J Infect.

[19] Shi H, Zuo Y, Yalavarthi S, Gockman K, Zuo M, Madison JA (2021). Neutrophil calprotectin identifies severe pulmonary disease in COVID-19. J Leukoc Biol.

[20] Ramlall V, Thangaraj PM, Meydan C, Foox J, Butler D, Kim J (2020). Immune complement and coagulation dysfunction in adverse outcomes of SARS-CoV-2 infection. Nat Med.

[21] Lupu F, Keshari RS, Lambris JD, Coggeshall KM (2014). Crosstalk between the coagulation and complement systems in sepsis. Thromb Res.

[22] Wool GD, Miller JL (2021). The impact of COVID-19 disease on platelets and coagulation. Pathobiology.

[23] Mannes M, Dopler A, Zolk O, Lang SJ, Halbgebauer R, Hochsmann B (2021). Complement inhibition at the level of C3 or C5: mechanistic reasons for ongoing terminal pathway activity. Blood.

[24] Distelmaier K, Adlbrecht C, Jakowitsch J, Winkler S, Dunkler D, Gerner C (2009). Local complement activation triggers neutrophil recruitment to the site of thrombus formation in acute myocardial infarction. Thromb Haemost.

[25] Foley JH, Walton BL, Aleman MM, O'Byrne AM, Lei V, Harrasser M (2016). Complement activation in arterial and venous thrombosis is mediated by plasmin. EBioMedicine.

[26] Ito T, Kakuuchi M, Maruyama I (2021). Endotheliopathy in septic conditions: mechanistic insight into intravascular coagulation. Crit Care.

[27] Goshua G, Pine AB, Meizlish ML, Chang CH, Zhang H, Bahel P (2020). Endotheliopathy in COVID-19-associated coagulopathy: evidence from a single-centre, cross-sectional study. Lancet Haematol.

[28] Ladikou EE, Sivaloganathan H, Milne KM, Arter WE, Ramasamy R, Saad R (2020). Von Willebrand factor (vWF): marker of endothelial damage and thrombotic risk in COVID-19?. Clin Med (Lond).

[29] Cugno M, Meroni PL, Gualtierotti R, Griffini S, Grovetti E, Torri A (2021). Complement activation and endothelial perturbation parallel COVID-19 severity and activity. J Autoimmun.

[30] Yu J, Yuan X, Chen H, Chaturvedi S, Braunstein EM, Brodsky RA (2020). Direct activation of the alternative complement pathway by SARS-CoV-2 spike proteins is blocked by factor D inhibition. Blood.

[31] Duerschmied D, Suidan GL, Demers M, Herr N, Carbo C, Brill A (2013). Platelet serotonin promotes the recruitment of neutrophils to sites of acute inflammation in mice. Blood.

[32] Del Conde I, Cruz MA, Zhang H, Lopez JA, Afshar-Kharghan V (2005). Platelet activation leads to activation and propagation of the complement system. J Exp Med.

[33] Hottz ED, Azevedo-Quintanilha IG, Palhinha L, Teixeira L, Barreto EA, Pao CRR (2020). Platelet activation and platelet-monocyte aggregate formation trigger tissue factor expression in patients with severe COVID-19. Blood.

[34] Yao Y, Cao J, Wang Q, Shi Q, Liu K, Luo Z (2020). D-dimer as a biomarker for disease severity and mortality in COVID-19 patients: a case control study. J Intensive Care.

[35] Osawa I, Okamoto K, Ikeda M, Otani A, Wakimoto Y, Yamashita M (2021). Dynamic changes in fibrinogen and D-dimer levels in COVID-19 patients on nafamostat mesylate. J Thromb Thrombolysis.

[36] Wijarnpreecha K, Ungprasert P, Panjawatanan P, Harnois DM, Zaver HB, Ahmed A (2021). COVID-19 and liver injury: a meta-analysis. Eur J Gastroenterol Hepatol.

[37] Baj J, Karakula-Juchnowicz H, Teresinski G, Buszewicz G, Ciesielka M, Sitarz E (2020). COVID-19: specific and non-specific clinical manifestations and symptoms: the current state of knowledge. J Clin Med.

[38] Cuschieri S (2019). The CONSORT statement. Saudi J Anaesth.

