# Supplementary material for: Complement system component dysregulation is a distinctive feature of COVID-19 disease: a prospective and comparative analysis of patients admitted to the emergency department for suspected COVID-19 disease
Source: J Thromb Thrombolysis. 2021 Dec 14;53(4):788–97. doi: 10.1007/s11239-021-02617-x (PMC8668393; doi:10.1007/s11239-021-02617-x)
Supplement: Supplementary file 1 — Supplementary file1 (DOCX 220 KB) [file 11239_2021_2617_MOESM1_ESM.docx]

Supplement

Figure 1: Composition of diseases represented in the non-COVID-19 cohort.


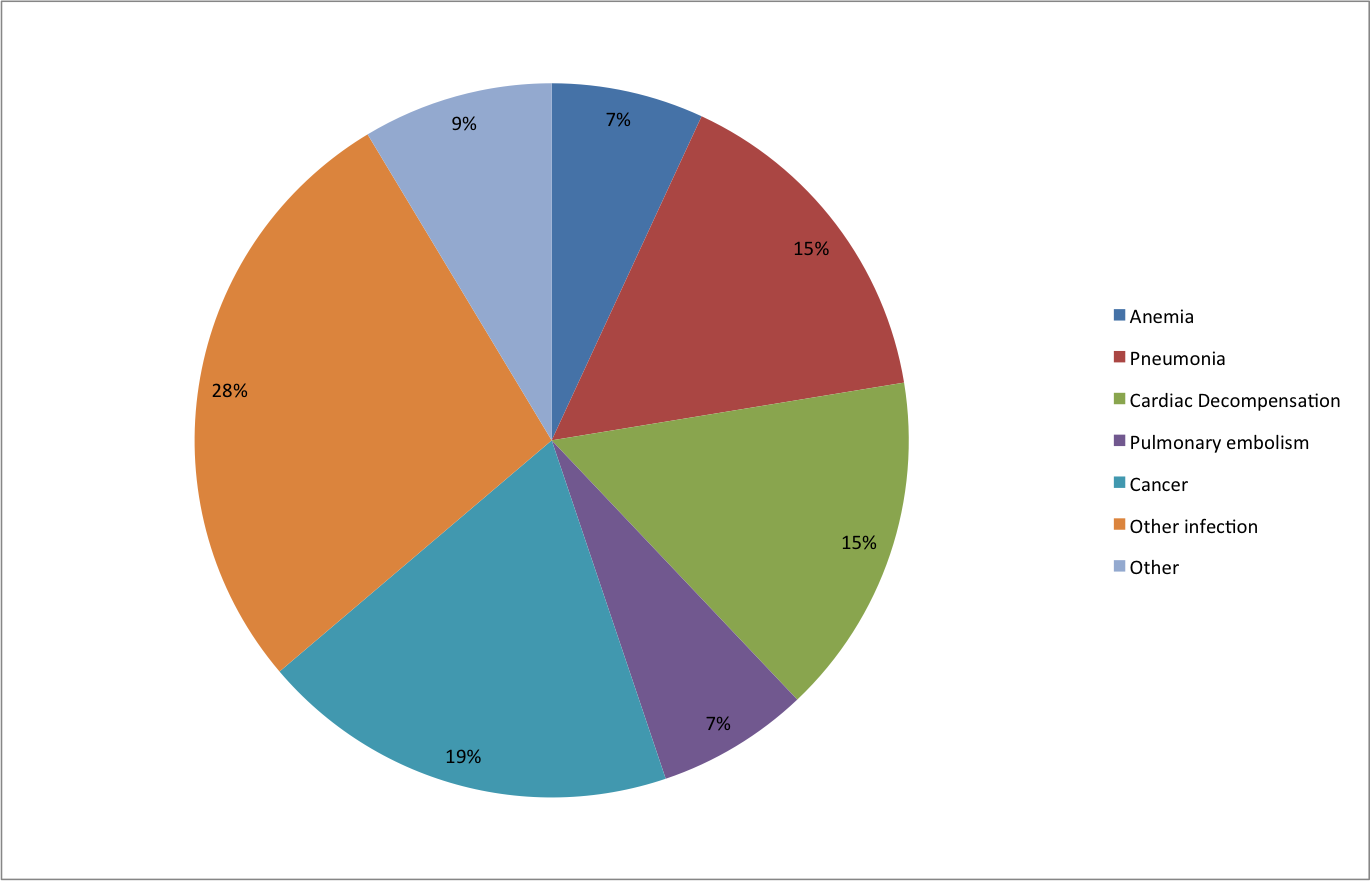


Figure 2: Comparative analysis of Complement factors (a: Complement component 4 in g/L; b: Complement component 3 in g/L) in patients hospitalized for suspected COCID-19 infection with SOFA Score >2 (n=14) and COVID-19 respectively. Data are presented as scatter block with median and interquartile range.

**a**


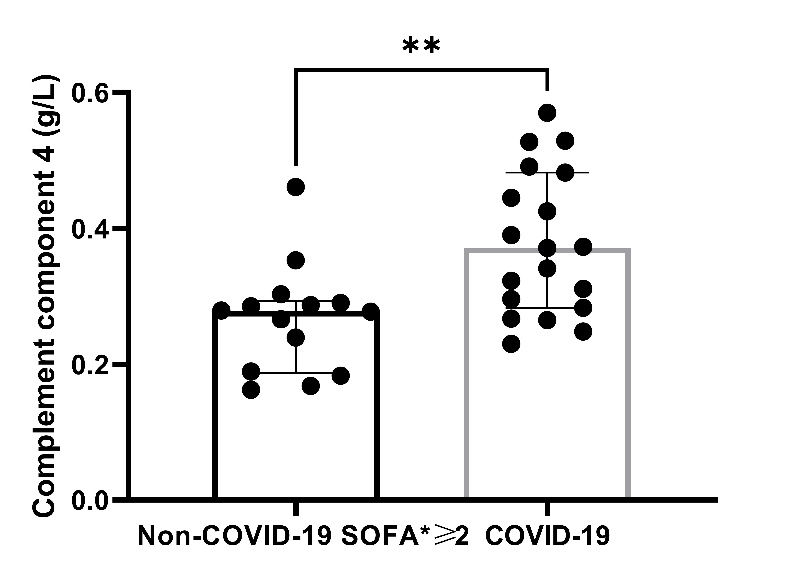


**b**


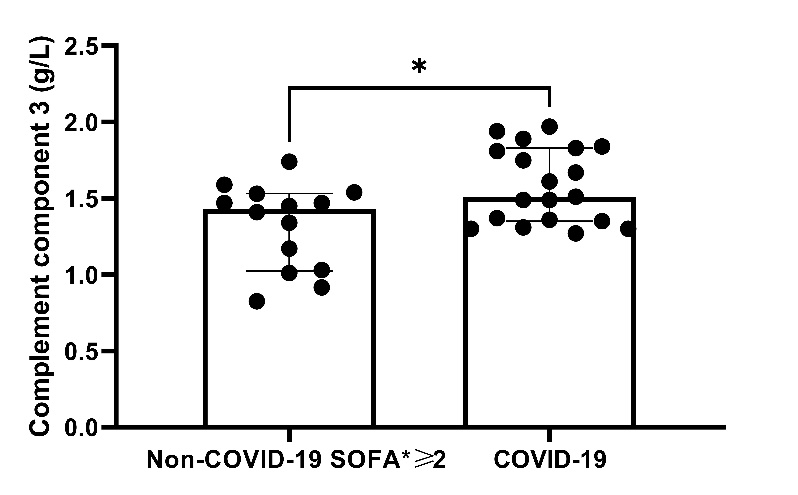


*SOFA= Sepsis-related organ failure assessment score
